# Editorial: Determinants of Synaptic Information Transfer: From Ca^2+^ Binding Proteins to Ca^2+^ Signaling Domains

**DOI:** 10.3389/fncel.2016.00069

**Published:** 2016-03-16

**Authors:** Philippe Isope, Christian D. Wilms, Hartmut Schmidt

**Affiliations:** ^1^Institut des Neurosciences Cellulaires et Integratives, Centre National de la Recherche Scientifique UPR 3212Strasbourg, France; ^2^Wolfson Institute for Biomedical Research and Department of Neuroscience, Physiology and Pharmacology, University College LondonLondon, UK; ^3^Medical Faculty, Carl-Ludwig Institute for Physiology, University of LeipzigLeipzig, Germany

**Keywords:** synaptic calcium signaling, calcium binding proteins, localization, calcium domains, transmitter release, methods

Ca^2+^ ions are key regulators of fundamental synaptic processes including transmitter release and the induction of plasticity. They act within complex topographical relationships between the sites of Ca^2+^ influx and those sites where the Ca^2+^ controlled effector proteins are located. These topographies are dynamically shaped by protein-complexes and the spatio-temporal extent of Ca^2+^ elevations within these topographies is controlled by Ca^2+^ buffers. Ultimately, these spatio-temporal relationships determine the details of Ca^2+^ induced effects. This e-book deals with the significance of localized synaptic calcium signaling.

Synaptic information transfer begins with presynaptic transmitter release. Wang and Augustine review the concept of local presynaptic Ca^2+^ signaling domains and their functional importance for release, focusing on two giant presynaptic terminals, the squid giant synapse and the calyx of Held. Central concepts that still dominate our view about the significance of presynaptic Ca^2+^ domains were originally developed using these two synapses. The authors describe the distinction between nano- and micro-domain topographies and the evidence for a developmental regulation of Ca^2+^ domains at several synapses.

A synapse that operates with nano-domain influx-release coupling is the parallel-fiber (PF) to Purkinje cell (PC) synapse. These synapses show use-dependent facilitation that surprisingly persists in mutant mice lacking calretinin—the major buffer of PF terminals—which leads to increased release probability. Brachtendorf et al. analyzed mechanisms of facilitation at individual PF-PC synapses of calretinin mutant mice using paired patch-clamp recordings. They suggest that a Ca^2+^-driven process that rapidly replenishes releasable vesicles operates more effectively in the absence of calretinin, thereby explaining the persistence of facilitation.

How critical the maintenance of calcium homeostasis on synaptic function can be is demonstrated by Orduz et al. In their study, knocking out calbindin, a calcium binding protein (CaBP) related to calretinin, had surprisingly little effect on the amplitude of postysynaptic potentials (PSPs) at PC-to-PC synapses. Their detailed study of presynaptic morphology revealed larger boutons and AZs and a higher number of docked vesicles in the mutants. The authors view these changes as a compensatory mechanism to maintain central characteristics of release in the face of a major perturbation.

For a given topology, CaBPs are key controllers of synaptic efficacy. Whether they simply act as buffers or have an additional sensor function is a critical question. Using constrained modeling, Timofeeva and Volynski shed light onto an often-overlooked candidate for the control of neurotransmitter release, Calmodulin (CaM). Acting as a buffer, CaM can influence calcium concentration near the AZ. The authors demonstrate that the fast calcium binding properties of CaM combined with its mobility inhibit vesicular release and favor short term facilitation during repeated action potential (AP) firing.

Many studies have established how CaBPs shape the calcium wave in the presynaptic bouton or the dendritic spines. Indeed, the mobility of CaBPs will determine how they influence calcium dynamics. Matthews and Dietrich review and discuss the role and the properties of immobile or poorly mobile buffers, demonstrating that they also participate in the control of the apparent diffusion of free calcium and its decay time constant.

The combination of calcium influx, extrusion, and buffering result in the calcium wave that triggers release De Jong and Fioravante review how this wave is translated into actual vesicular release. They described the mechanisms by which the calcium sensors expressed in the presynaptic compartment undergo conformational changes when calcium binds to specific domains and the role of specific candidates in the control of vesicular release.

A calcium sensor central to synaptic release is synaptotagmin. Using a combination of electro-physiological recordings and super-resolution microscopy, Paul et al. reveal an unexpected interaction between the AZ protein Bruchpilot and synaptotagmin. They find that in *Drosophila* differentiation of neuromuscular junctions require concerted action of both Bruchpilot and synaptotagmin to result in structurally and functionally normal synapses. This study exemplifies how the combination of physiology and high-resolution microscopy can be harnessed to elucidate synaptic function at the nanometer scale.

Estimates of nano-scale topographical relationships at AZs are often derived by combining quantitative physiological techniques with modeling approaches. However, as Ehmann et al. point out in their Perspective, without direct microscopic access to the synaptic fine structure, our picture of the function of the surrounding protein networks will remain incomplete. Due to the diffraction-limit for light investigations of the AZ ultrastructure have been restricted to EM, which however provides only a static snap-shot. The authors explore, how recent advances in super resolution light microscopy like STED and STORM bridge the gap between EM and conventional light microscopy.

Next to AP triggered release, vesicle exocytosis also occurs spontaneously at most synapses. Truckenbrodt and Rizzoli review the controversy about the mechanism of spontaneous exocytosis. It is thought that either spontaneous fluctuations in local calcium trigger fusion of vesicles residing in the same vesicle pool that normally fuses during an AP albeit via a different release sensor, or else that release is driven from a separate pool of synaptic vesicles probably independent of Ca^2+^. The authors speculate on the origins of this controversy and suggest that a potential solution could be related to developmental effects.

Neurotransmitter release is highly influenced by the structural organization of calcium channels. Notably, the distance and the spatial organization between synaptic vesicles and calcium channels affect the probability of release. Using high-resolution freeze fracture immuno-EM and automatized reconstruction of Cav2.1 channel distribution, Althof et al. demonstrated that inhibitory and excitatory synapses in the CA1 area of the hippocampus share a common spatial organization.

While the papers above focus on the role of calcium topography and dynamics *in the presynaptic compartment*, the central role of these parameters is of course not limited to the presynapse.

The amplitude and time course of dendritic calcium transients are not only determined by buffering and extrusion, but also by morphology, specifically the dendritic diameter. Anwar et al. explore the expected variance of calcium concentrations due to differences in dendritic diameter using a modeling approach. In addition the authors demonstrate how to implement diameter-dependent effects on calcium signaling in the NEURON simulator. Armed with this knowledge researchers will be able to include such strong morphological effects in their models of dendritic function.

To close the cycle of information flow, PSPs generated by synaptic inputs are conducted to the soma. How strongly these PSPs are filtered and attenuated is to a larger part determined by the electrotonic compactness of the dendritic branch. Using whole-cell recordings directly from the dendrites of cerebellar granule cells (GCs), Delvendahl et al. analyze the electrotonic compactness of these minute neurons. They find that GCs are electrotonically extremely compact, making them ideally suited for the efficient transfer of high-frequency information.

This research topic gives a prospect onto how the multitude of synaptic Ca^2+^ functions arises from the localization of Ca^2+^ signals to specific sub-compartments by the joined action of topographical relationships with CaBPs and emphasize the importance of technical breakthroughs in promoting our understand compartmentalization.

## Author contributions

All authors listed, have made substantial, direct and intellectual contribution to the work, and approved it for publication.

## Funding

This work was supported by an ARN grant to PI (FRM 2014 DEQ20140329514) and a DFG grant to HS (SCHM1838).

### Conflict of interest statement

The authors declare that the research was conducted in the absence of any commercial or financial relationships that could be construed as a potential conflict of interest.

